# When music is salty: The crossmodal associations between sound and taste

**DOI:** 10.1371/journal.pone.0173366

**Published:** 2017-03-29

**Authors:** Rachel Guetta, Psyche Loui

**Affiliations:** 1 Department of Psychology, Wesleyan University, Middletown, Connecticut, United States of America; 2 Program in Neuroscience & Behavior, Wesleyan University, Middletown, Connecticut, United States of America; University of Zurich, SWITZERLAND

## Abstract

Here we investigate associations between complex auditory and complex taste stimuli. A novel piece of music was composed and recorded in four different styles of musical articulation to reflect the four basic tastes groups (sweet, sour, salty, bitter). In Experiment 1, participants performed above chance at pairing the music clips with corresponding taste words. Experiment 2 uses multidimensional scaling to interpret how participants categorize these musical stimuli, and to show that auditory categories can be organized in a similar manner as taste categories. Experiment 3 introduces four different flavors of custom-made chocolate ganache and shows that participants can match music clips with the corresponding taste stimuli with above-chance accuracy. Experiment 4 demonstrates the partial role of pleasantness in crossmodal mappings between sound and taste. The present findings confirm that individuals are able to make crossmodal associations between complex auditory and gustatory stimuli, and that valence may mediate multisensory integration in the general population.

## Introduction

Audition and gustation are two complex sensory and perceptual experiences. While hearing and tasting are both universal biological systems, they are also strongly influenced by culture and learning. Rather than considering these sensory modalities as separate entities, here we explore if, and to what extent, humans form associations between complex sound and complex taste, and what mechanism may underlie these crossmodal experiences. Indeed, we know that our senses work together and can simultaneously receive information about the same external stimulus or event. Our gustation faculties intertwine with our sense of smell to produce taste, and we constantly fuse input from multiple senses to analyze our environment [1–4].

Even when multisensory experiences are automatic, as in the case of synesthesia, much work suggests that synesthetes rely on broader cognitive mechanisms and that these associations may generalize to a greater population [5–7]. Crossmodal associations, then, may represent normal mechanisms that link the perception of one modality to another, integrating faculties such as attentional binding, linguistic thought, learning and memory, and preferences for pleasantness [8]. We hope that a deeper understanding of these mechanisms can inform how and why dramatic individual differences in perception exist.

Crossmodal correspondences have been reported between most combinations of visual, auditory, tactile, olfactory and gustatory stimuli [9]. Researchers have recently begun to explore the everyday nature of crossmodal associations between sound and taste [10–14]. Music can be evaluated as sweet, sour, salty or bitter, depending on features in its composition, such as pitch, articulation, or brightness [15–17]. Higher pitches are often evaluated as sweet or sour, while lower pitches tend to correspond with bitterness, thus mapping perceptual associations between the two modalities [10, 15]. Associations between sound and taste have been reported mainly in the context of relative pitch, instrument timbre and taste words, wherein participants map simple auditory stimuli to semantic representations of taste words [10, 13, 15]. In one study musicians and non-musicians, when prompted by one of the four taste labels (sweet, sour, salty or bitter), were instructed to improvise a composition on a MIDI keyboard in response to the label [13]. “Bitter” triggered lower pitches and legato notes, whereas “salty” triggered staccato articulation, “sour” resulted in dissonance, and “sweet” led to consonance and slower and softer notes. Furthermore, naïve listeners could easily guess which taste label had triggered the improvisation upon listening to the clips, thus revealing a symmetrical mapping between music and semantics. Other studies have shown a similar correlation, using somewhat more complex stimuli. Participants presented with flavored milk solutions of varying fat content were asked to match milk samples to pitches [11]. They were consistently able to choose both the pitch class and the instrument they felt best matched each sample regardless of fat content.

Taken together, evidence suggests that the general population is able to associate sound and taste based on a match between musical parameters and gustatory properties. Behind the intricate compilation of pitch, harmony, articulation and tonal patterns, and behind the conglomeration of sugars, acids, nutrients and proteins, the normal brain is able to connect the two modalities by extracting meaning, similar to results from priming studies between music and language [18]. Although reliable crossmodal correspondences have been observed in the broader population, the explanation for these associations has yet to be determined.

Several hypotheses debate whether crossmodal associations are universal and innate [19– 20] or learned from development [14, 21–22], impacted by culture or region [23] or influenced by subjective preference or pleasantness [24–26]. More generally, some claim that perception is inherently synesthetic for all infants, and that crossmodal associations are an innate property of perception [27–28]. Preferential looking tasks in 3- to 4-month-old preverbal infants [29] have shown sensitivity to correspondences between auditory pitch and visuospatial height: infants looked longer at a changing visual display when accompanied by a sound whose changing pitch was congruent to the height change, supporting crossmodal associations as a fundamental aspect of perception. On the other hand, there is support for crossmodal associations as a result of statistical co-occurrences. There is evidence that newborns of different mammalian species protrude their tongues upwards in response to pleasant tastes and downwards in response to aversive tastes, which co-occurs with high and low pitches, respectively, when exhaling air [12, 20]. By this account, infants encode the co-occurrences of their innate orofacial gestures and the resulting auditory cues.

The cultural hypothesis has garnered recent support, as well. 452 participants from China, India, Malaysia, and the U.S. matched colors, shapes, and textures to taste words, making nonrandom crossmodal correspondences between certain colors and shapes and bitter, sour and sweet taste words. Cross-cultural similarities and differences were observed in taste-patterns across the four countries: participants from all four groups associated salty tastes with the color white, sour tastes with the color green, and sweet tastes with the color pink. Conversely, the color blue was interpreted similarly to the color white by the Malaysian, Chinese and Indian participants, whereas this trend was nonexistent in participants from the U.S., and Indian and Chinese participants categorized colors in different ways, thus providing support for the role of culture in sound-taste associations [23].

Finally, the explanation that positive and negative valence of auditory and gustatory stimuli may drive crossmodal associations is not an altogether novel concept. One study showed that ratings for simple perceptual stimuli across various studies were reducible to a two-dimensional emotion space of valence and activity, providing support for an emotional basis for responses to perceptual stimuli [24]. The valence-matching hypothesis has garnered support from much research [25–26, 30–33], with results generally showing that participants match tastes that are perceived to be unpleasant (e.g., bitter) with sounds that are similarly unpleasant (e.g., trombone sounds), and more pleasant tastes (e.g., sweet) with more pleasant sounds (e.g., piano sounds) [15, 30].

However, while studies demonstrate mapping between simple auditory and gustatory stimuli, direct evidence of crossmodal correspondences between complex sound and taste stimuli is sparse. There is a growing need for researchers to incorporate complex and naturalistic stimuli in taste and gustation research [34]. And while some more recent studies have paired more complex taste with simple auditory stimuli, such as musical pitch or timbre, there are few studies examining the matches between both complex sound and complex taste.

Here, we investigate the presence of crossmodal associations between complex sounds and complex tastes, and the role of preference as a mediator of these associations. The auditory stimuli consisted of recordings of an original violin composition, as opposed to isolated pitches or timbres. The gustatory stimuli consisted of custom-made chocolate ganache instead of simple stimuli, beverages, or merely the names of food or flavors. We chose chocolate, as it can adapt to sweet, sour, salty and bitter tastes, and because it can elicit a wide range of pleasantness ratings. And while past studies have used isolated, basic taste samples of flavored beverage solutions, chocolate is a more naturalistic gustatory stimulus. Both violin music and chocolate ganache are categories of complex stimuli that enable fine-grained perceptual discrimination, providing a novel set of stimuli with which to investigate crossmodal associations.

Although the literature on complex sound-taste associations is varied, the cultural and statistical co-occurrence hypotheses may predict an effect of musical training, as musicians can be expected to have more cultural and statistical exposure to sound features that map onto crossmodal associations. Thus, those with more extensive musical training may experience a more keen awareness of articulation and textural changes between musical stimuli, which, in turn, engender stronger associations. In contrast, the emotional valence hypothesis may predict stronger crossmodal experiences for more valenced stimuli. That is, more pleasurable (positively-valenced) music and taste stimuli may elicit stronger crossmodal associations overall. The emotional valence hypothesis may also predict differences in crossmodal mappings between participants with varying preferences of gustatory and auditory stimuli.

## Experiment 1

The first experiment validates our auditory stimuli, and tests whether participants without any prior exposure to the musical stimuli are able to match the music clips to the intended taste labels.

### Materials and methods

#### Participants

160 Amazon Mechanical Turk workers, all from the U.S., completed a total of 160 ratings. Each participant was compensated $0.15. Subjects were unselected for gender, age, ethnicity, and musical training.

#### Stimuli

Auditory stimuli consisted of eight recordings of violin music. An original melody was composed for this experiment in order to minimize any rote memory, familiarity, or associations from known pieces. The eight clips were comprised of one melody played in four different styles, each style with two versions (that is, two versions of a sweet clip, two versions of a sour clip, etc.). Each of the four styles incorporated different musical articulation to mimic the connotations of sweet, sour, salty and bitter as informed by prior studies. Previous findings have shown that different flavors are associated with different timbres and articulations of musical notes: bitter triggers more low-pitched and legato notes; salty connotes staccato articulation; sour results in dissonance; and sweet is associated with consonant, slow and soft notes [10–11, 12–13, 15, 30]. Our sweet clip, then, is legato, with more vibrato and emphasis on the higher notes. The salty clip is played at a quick tempo with staccato articulation. The bitter clip has a richer tone at a slower tempo, with vibrato and emphasis on the lower notes. The sour clip incorporates dissonance and slight glissandos.

The consistent use of the violin throughout all eight music clips, and the sameness in melody, assures experimental control of pitch and instrumentation as well as melody and implied harmony, and minimized possible associations between long-term experience and musical structure. The clips range in duration between 10 and 22 seconds, based on the various tempos to achieve four distinct styles of sweet, sour, salty and bitter music. The eight recordings are available online at https://wesfiles.wesleyan.edu/home/ploui/web/SoundTasteCrossmodal/.

#### Procedure

Participants first listened to a single music clip, and then selected a taste word (sweet, sour, salty or bitter) that best matched the clip. Participants were allowed one minute to complete the assignment. Each of the 160 participants only listened and responded to one music clip, as to ensure that each musical stimulus could be interpreted even without the context of the other three styles. In this way, participants’ responses also helped to test the efficacy of the stimuli.

Twenty ratings were obtained for each of the eight music clips.

#### Data analysis

A response was considered correct if the participant’s chosen word matched the target taste category of the music clip. One participant’s data was excluded because they did not listen to the clip in its entirety.

### Results

A chi-square test for association, with cell arrays organized by each of the eight music clips and each of the four taste labels, is significant, χ²(21, N = 159) = 60.17, p < 0.001, confirming that there was an association between music clips and chosen taste labels. Participants performed significantly above chance level of 0.25 (M = 0.46, SE = 0.04, t(158) = 5.28, p < .0001) when matching the musical stimuli to corresponding taste labels. Across all four categories of musical stimuli, participants were able to identify the sour clips with greatest accuracy (sour = 0.51, bitter = 0.48, sweet = 0.45, salty = 0.4), but differences in accuracy between the categories of tastes were not significant, as reflected in a one-way ANOVA comparing the four taste groups (F(3,158) = 0.35, n.s.). Accuracy for sour and bitter categories were significantly above chance at the p < .05 level even after Bonferroni correction for multiple comparisons, as shown in Table 1.

**Table 1:** Means, standard errors, and one-sample t-test against chance level of 0.25 for each taste category from Experiment 1.

Taste Category	N	Mean	Std. Error Mean	t	df	p (2-tailed)	p (Bonf. corrected)
Bitter	40	0.475	0.07996	2.814	39	0.008	0.032
Salty	40	0.4	0.07845	1.912	39	0.063	n.s.
Sour	39	0.5128	0.08108	3.241	38	0.002	0.008
Sweet	40	0.45	0.07966	2.511	39	0.016	0.064

### Discussion

Despite no prior exposure to the musical stimuli, and no explicit training of the associations between sound and taste, participants were able to match the clips to taste words with above-chance accuracy. This suggests that individuals can form associations between complex auditory sensations and simple taste labels. The findings also confirm the efficacy of our auditory stimuli in eliciting matches with sweet, sour, salty and bitter taste groups. The following experiments investigate crossmodal associations between complex auditory and complex gustatory stimuli, and explore the mechanism that may contribute to such experiences.

## Experiment 2

Before examining the associations between complex sound and taste, it is important to consider the auditory, acoustical parameters that may influence participants’ perceptions and categorizations of the music clips. We know that taste has different dimensions, and that flavors cluster together [35]. The question we try to answer here, by means of multidimensional scaling (MDS), is if analogous dimensions can organize musical stimuli as well. MDS allows us to reduce the multidimensional space of musical articulation into two or three dimensions. By collapsing these dimensions, we may begin to draw inferences about higher-level descriptors and possible perceptual or cognitive continua that may mediate complex crossmodal associations between sound and taste.

### Materials and methods

#### Participants

16 Wesleyan University undergraduate students participated in this experiment (4 male, 12 female) for $10 per hour. All participants reported that they had normal hearing, and no impairment of smell or taste functions. Number of years of musical training ranged from 0 (no musical training) to 15 years (M = 6.4, SE = 1.33). The heterogeneous sample in terms of musical training allows for a spectrum of experience, and a way to analyze any specific trends in how training may affect crossmodal associations. Participants’ instruments included guitar, piano, flute, cello, trombone, bass, drums and vocal training. All participants gave written informed consent before the experiment, as approved by Wesleyan University’s Department of Psychology Ethics Committee.

#### Stimuli

The same eight music clips were used (two samples of each musical category: sweet, sour, salty and bitter). The test was administered using custom software written in Max/MSP [36].

#### Procedure

Each trial consisted of two randomized music clips played consecutively. Twenty-eight total trials of pairwise similarity ratings were administered to account for all possible pairs within the eight music clips (8 choose 2 = 28). After each pair of stimuli, participants rated how similar or different the two stimuli were from each other on a slider-controlled visual analog scale from “very different” to “very similar.” The position of the slider on the scale was converted to a number from 1 (most different) to 100 (most similar) for analysis.

#### Data analysis

Pairwise ratings were averaged across participants and normalized to an 8 by 8 distance matrix with values between 0 (most similar) and 1 (most different). Classical multidimensional scaling was performed on the similarity matrix in Matlab with the classical multidimensional scaling [37] with two dimensions specified in the output. A scree test showed that two dimensions were adequate in representing the variables (stress value = 0.089, R^2^ = 0.9726).

### Results

The multidimensional scaling solution from pairwise similarity ratings generated a pattern in which the two music clips within the same taste category yielded “very similar” ratings, whereas clips from different taste categories garnered “different” ratings. This further supports the efficacy of our auditory stimuli. Participants were also able to clearly distinguish differences between music clips within the four distinct taste categories. Within each category, average similarity ratings differed: ratings were highest for sweet and salty pairs, and lowest for sour and bitter pairs (F(3,45) = 4.3, p = .009). These differences may reflect acoustic features that are easier to detect within the salty pair (staccato articulation) and the sweet pair (legato articulation).

The two-dimensional multidimensional scaling solution shows clear clustering of the musical stimuli by taste categories. The first dimension discriminates the salty stimuli from the rest of the stimuli, whereas the second dimension, which captures variability that is unexplained by the first dimension, distinguishes among the sweet, bitter, and sour stimuli.

**Fig 1:**
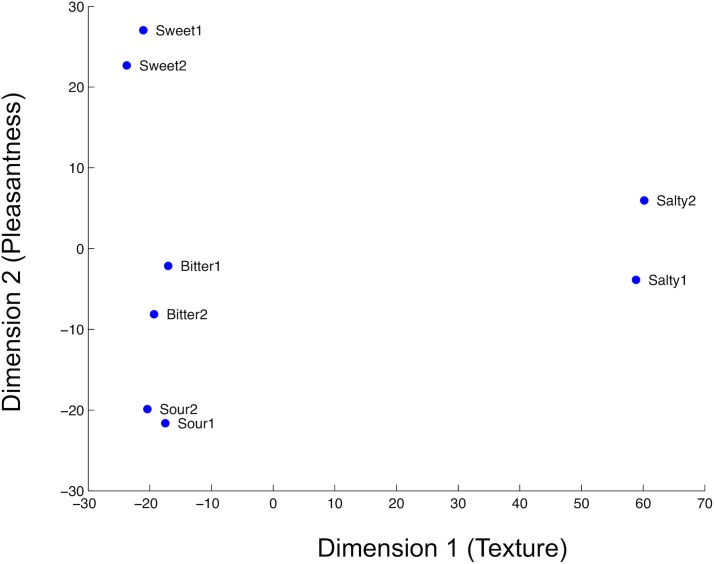
Two-dimensional representation of participants’ similarity ratings of the complex auditory stimuli. The model shows clustering of individual musical stimuli by corresponding tastes. Each point on the space corresponds to one auditory stimulus, labeled with the associated taste. Dimension 1 is interpretable as texture and dimension 2 as pleasantness.

Based on these findings we interpret the two dimensions to reflect “texture” (see Dimension 1 in Fig 1) and “pleasantness” (see Dimension 2 in Fig 1). Together, the two dimensions explain over 97% of the total variance among average ratings for all eight musical stimuli.

### Discussion

The MDS technique illustrates how participants represent complex auditory stimuli, consistent with taste dimensions. As expected, the two music clips recorded within the same taste category are close in proximity. The style of musical playing–articulation, nuances, accents, richness–all play a role in distinguishing the stimuli from one another. In addition to the melody itself, which remains the same in all eight clips, these characteristics are the only differences between the clips and therefore influence how similar or different the stimuli are from each other. These attributes of texture, then, contribute to Dimension 1. The salty stimuli appear furthest from the rest, perhaps due to the stark differences in texture with a noticeably quicker tempo and a stickier, staccato articulation. The stimuli that shared common textural attributes, however, were rated as similar and are displayed in close proximity on the two-dimension solution. The sweet, sour, and bitter stimuli were all considerably more legato than the salty stimuli, which can explain their proximity along Dimension 1.

Dimension 2 is interpreted to reflect pleasantness, or the positive or negative content of the stimuli. Participants responded differently to the four musical styles, likely a result of the timbral differences known to affect listeners’ basic organization of musical [38]. Participants reacted most pleasantly to the sweet stimuli, with its warmth, vibrato and legato nature. The sweet clips were played with the least intensity in bow pressure, which is related acoustically to less loudness and less brightness, and slower attack and decay times [39]. The bitter and sour stimuli, however, were played with more tension and roughness with quicker fluctuations in amplitude envelope, eliciting a less pleasant reaction [40]. This clarifies the distance between the sweet stimuli from the rest. The sour stimuli accentuated dissonant intervals and emphasized proximal frequency components, creating a categorically unpleasant response, which can explain why the sour stimuli are rated lowest on Dimension 2. The salty stimuli with staccato articulation and quicker tempo are energetic and light, with quick attacks and releases of notes, and can be relatively pleasant, which may explain the relatively higher position on Dimension 2 [41]. The timbral differences amongst the four melodies, with discrete combinations of perceptual dimensions, created here a valence hierarchy. Pleasantness, with its approach/avoidance quality, is a prominent and intuitive reaction to all types of stimuli, and may therefore work as a link between sensory modalities. Hence, we interpret the two dimensions on our MDS model as texture and pleasantness.

## Experiment 3

Experiment 3 further investigates the crossmodal associations between sound and taste, specifically using chocolate as taste stimuli to investigate its mapping with musical stimuli.

### Materials and methods

#### Participants

50 Wesleyan University undergraduate students took part in the experiment (25 male, 25 female). Number of years of musical training ranged from 0 (no musical training) to 14 years (mean = 4.55, SE = 0.70). 16 were musicians (at least 7 years of musical training) and 34 were non-musicians. All participants reported that they had normal hearing, and no impairment of smelling or taste functions. All participants gave written informed consent before the start of the experiment, as approved by Wesleyan University’s Department of Psychology Ethics Committee.

#### Stimuli

Custom-made chocolate ganache was used as gustatory stimuli, as it can adapt to the four basic taste groups. The sweet ganache was made with 61% semisweet cacao chocolate; the sour ganache was made with 1.5 tablespoons of white distilled vinegar per 10 ounces of 61% semisweet cacao; the salty ganache was made with 1 teaspoon of salt per 8 ounces of 61% semisweet cacao; and the bitter ganache was made with 100% bittersweet cacao. Each batch was made with a ratio of 10 ounces of chocolate to 6 ounces of heavy whipping cream. Each chocolate had the same color and consistency.

As a control task, 19 different participants sampled all four flavors, assigned each sample a taste label, and rated the strength of each flavor on a continuous visual analog scale of 1–6. Ratings were normally distributed and similar across flavors (Sweet: M = 4.41, SE = 0.23; Sour: M = 4.05, SE = 0.31; Salty: M = 4.08, SE = 0.32; Bitter: M = 4.48, SE = 0.85). A one-way ANOVA comparing these four strengths was not statistically significant (F(3,66) = 1.08, p = 0.36). Thus, the four different tastes were psychophysically matched in strength of flavor.

The auditory stimuli used in this experiment were the same violin recordings used in the previous experiments, and were presented via a computer interface written in Max/MSP [36].

#### Procedure

Participants were first presented with a familiarization phase, in which they sampled each of the four chocolates and listened to each of the four musical stimuli, all in randomized order. Next, participants were tested for their associations between the music and the chocolate. Five trials were presented to eliminate selection by process of elimination, while minimizing fatigue and habituation from repeated stimulus presentations. For each trial, participants ate a randomly assigned piece of chocolate, then listened to each of the four music clips in their entirety. The task was then to select which music clip best fit the chocolate they had just sampled. Participants were free to listen to the clips as many times as they wished before making their selection. As a palate cleanser between successive trials, participants were offered a bite of pita bread. No taste words were introduced while testing for the crossmodal association itself in order to avoid any semantic influences.

#### Data analysis

A response was considered correct if the participant’s chosen music clip matched the target taste of the chocolate ganache.

### Results

A chi-square test for association between the four chocolate ganaches and the four music clips is significant, χ²(9, N = 234) = 36.7, p < 0.001. Participants matched each ganache to the respective music clip (that is, the sweet ganache to the sweet music clip, the sour ganache to the sour music clip, etc.) above chance level of 0.25 (M = .35, SE = 0.04, t(49) = 2.59, p = .013). A one-way ANOVA comparing the four taste groups was not statistically significant, but showed a trend towards possible differences across the taste groups (F(3,196) = 2.2, p = .08). The sweet match rate was the strongest (M = 0.49, SE = 0.07), followed by salty (M = 0.38, SE = 0.07), bitter (M = 0.30, SE = 0.06) and sour (M = 0.29, SE = 0.06). While overall average accuracy was above chance level of .25, separately comparing match rates for each taste group showed significantly above-chance performance for the sweet category only (t(49) = 3.40, p = .001), while performance for the salty category showed a trend (t(49) = 1.88, p = .067) and the sour and bitter categories were not significantly above chance (sour t(49) = .64, bitter t(49) = .783, both n.s.). Musically trained participants (M = 0.33, SE = 0.07) did not perform more accurately than non-musically trained participants (M = 0.36, SE = 0.05, F(1,48) = 0.11, n.s.), and accuracy did not correlate with number of years of musical training (Pearson r = -.02; Spearman rank-order correlation r_s_ = -.05; both n.s.). The effect of sex on accuracy was not significant, as shown by an independent-samples t-test comparing accuracy by male and female participants (t(48) = 1.54, n.s.).

### Discussion

Participants were able to associate across sound and taste modalities. This is a first demonstration of sound-taste matching with both complex sound and complex taste. Although matching performance was significantly above chance level, match accuracy was not highly accurate due to considerable variability among the participants, which was not explained by musical training. This variability suggests some mediating factors between sound and taste perception that might explain individual differences.

## Experiment 4

While we observed significant associations between complex sound and complex taste stimuli, the possible mechanisms that might mediate these experiences are yet unclear. Previous work on sound-taste association has suggested the use of emotional valence of sound and taste stimuli as a feature that is shared between the two sensory modalities. The use of preference as a cue was also confirmed in preliminary verbal interviews with participants. Experiment 4 thus explores the mediating role of preference in sound-taste crossmodal associations.

### Materials and methods

#### Participants

23 Wesleyan University undergraduate students (15 male, 8 female) who took part in the previous experiment additionally participated in this follow-up study. Within this sample, 8 were musicians (at least seven years of musical training) and 15 were non-musicians. Number of years of musical training ranged from 0 (no musical training) to 19 years (M = 3.83, SE = 1.07). The current sample of participants (in Experiment 4) did not differ from the overall sample of participants (from Experiment 3) in musical training (t(48) = 1.19, p = 0.12) or in accuracy (t(48) = 0.83, p = 0.21) of the matching task in Experiment 3.

#### Stimuli

The same custom-made chocolate ganache as was used in Experiment 3 was used here, all four with the same color and consistency and psychophysically matched in strength of flavor. The auditory stimuli used in this experiment were the same violin recordings used in the previous experiments, and were presented via a computer interface written in Max/MSP [36].

#### Procedure

Participants were asked to rate each music clip and each ganache flavor on a 7-point scale, anchored by “highly unpleasant” (1) and “highly pleasant” (7).

#### Data analysis

Among the 23 participants 6 preferred the sweet music clip, 6 preferred the salty clip, 2 preferred the sour clip, and the remaining 9 participants gave similar preference ratings for two or more categories. No participants preferred the bitter music clip. Based on these music preference ratings we grouped the participants into sweet, salty, and sour music preferrers, and further identified taste preference profiles of each group. 14 participants preferred the sweet chocolate, 2 participants preferred the salty chocolate, 1 participant preferred the bitter chocolate, and the remaining 6 participants gave similar preference ratings for two or more ganache samples.

### Results

Given the 7-point scale, average pleasantness ratings for the ganache samples ranged from 3.70 (SE = 0.33) for bitter samples to 5.89 (SE = 0.24) for sweet samples. Sour samples averaged 4.18 (SE = 0.36) and salty samples averaged 4.06 (SE = 0.37). Pleasantness ratings for music were also lowest for bitter (M = 3.70, SE = 0.31) and highest for sweet (M = 4.90, SE = 0.37), with sour (M = 4.24, SE = 0.30) and salty (M = 4.86, SE = 0.33) in between. To assess the possible association between pleasantness ratings for the two modalities, we tested for a correlation between preferences for each musical stimulus and ganache sample. A significant positive correlation was observed between pleasantness ratings of gustatory and auditory stimuli across all trials (r(90) = .41, p < .0001). This correlation indicates an association between pleasantness ratings for the music clips and their corresponding ganaches: e.g. while most participants preferred sweet music and sweet chocolate overall, a participant who rated the salty ganache stimulus as pleasant was also likely to find the salty music clip to be pleasant. This similarity between pleasantness ratings across modalities suggests that participants could have associated sound and taste based upon their personal preferences.

**Fig 2:**
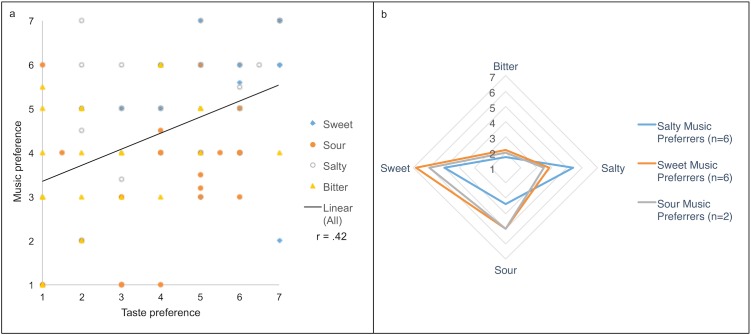
Taste preference mirrors music preference. (a) Correlation between preference ratings for music clips and ganache samples. (b) Taste preference profiles of sweet, sour, and salty music-preferring groups, showing that taste preference mirrors music preference. Radial axes represent taste preference ratings, and show that individuals preferring the sweet music clip also preferred the sweet ganache, while those preferring the salty music clip also preferred the salty ganache.

Fig 2A shows a correlation between preference ratings for music and chocolate samples, whereas Fig 2B shows taste preference profiles for sweet, sour, and salty music preferring subjects. Although participants generally preferred the sweet and not the bitter stimuli in both modalities, those who preferred sweet music stimuli also preferred sweet taste stimuli, whereas those who preferred salty music stimuli also preferred salty taste stimuli, highlighting the similarity between music and taste preferences.

### Discussion

While Experiment 3 shows that individuals are able to map sweet, sour, salty and bitter across taste and sound modalities, Experiment 4 explores a mechanism contributing to this effect. Pleasantness ratings suggest that emotional valence may act as one mediating factor in the crossmodal relationship between sound and taste. The similarity of preference ratings between sound and taste suggests that participants may match complex sounds and complex tastes based on their individual preference profiles that transcend sensory modalities.

## General discussion

The present work demonstrates and examines the crossmodal associations between sound and taste. Experiment 1 replicated the phenomenon that individuals can associate between complex sound and simple taste stimuli. We found that participants are able to match isolated, unfamiliar music clips with corresponding taste labels above chance level. Next, Experiment 2 used a multidimensional scaling method to reveal participants’ categorizations of sweet, sour, salty and bitter music clips. The MDS solution suggests that individuals are most influenced by texture and pleasantness of auditory stimuli. And while the majority of existing literature examines mapping between both simple auditory and gustatory stimuli, participants in Experiment 3 match complex sound to complex taste with above chance level accuracy. Finally, Experiment 4 provides support for emotional valence as a possible mediator of crossmodal associations. Results show that when participants successfully associate these complex sound and taste categories, there is also a highly significant correlation between pleasantness ratings in the two modalities.

This is one of the first studies to integrate complex auditory stimuli in a crossmodal matching paradigm, informed by the acoustical parameters and reciprocal mapping techniques across gustation and audition [12, 42], while maintaining the natural complexity and ecological validity sparse in existing studies [34]. Still, we implement a paradigm and methods similar to existing studies in testing the phenomenon of crossmodal associations, as well as the contributing mechanisms [11, 13, 42].

Our findings suggest that perhaps everyone, to some extent, has the capacity to form mappings between auditory and gustatory modalities. Although the role of past experience with sound (as embodied by musical training and experience) might be hypothesized to lead to increased ability to associate sounds with corresponding tastes, we tested this hypothesis by comparing participants with and without musical training. Results show that individuals with musical training were not more accurate in their sound-taste associations, suggesting that long-term experience with stimuli in one of these modalities is unlikely to affect associations with the other modality.

The present results further provide support for the role of emotional valence of stimuli in in mediating crossmodal mappings between sound and taste. Further studies can continue to parse specific acoustic features that contribute to such emotional responses, i.e. tension and relaxation. Future work may also explore how and to what extent the alternative hypotheses (i.e. the role of culture, learning and memory) might be at play as well. Additionally, it is possible that the preference ratings of the musical and gustatory stimuli in Experiment 4 reflected an element of learned association, as all participants had previously participated in crossmodal mapping in Experiment 3. As these explanations may not be mutually exclusive, the interrelatedness of structural, statistical and semantically mediated associations is still unclear. Further, despite our current results showing no significant sex differences in crossmodal associations, it is possible that biological sex contributes to individual variability. For example, taste sensitivity and olfactory thresholds vary depending on menstrual stage, which could affect crossmodal experiences [43].

Applications of these findings may apply to food businesses and restaurant entrepreneurs in marketing products and optimizing consumer experience, capitalizing on emotional congruency between sound and taste. Still, there is a need to better understand the interplay of neural correlates of emotional response during crossmodal experiences. Lastly, and most basically, there remains the question of why crossmodal associations between sound and taste exist in the first place.

While we recognize the strengths and implications of our integrative approach of using complex sound and taste, we also acknowledge the limitations of our methodology. Because we used different chocolate bases (three ganache samples with 61% cacao and one sample with 100% cacao), it is possible that the variance in fat content could have altered participants’ perception of stimuli, as it has been posited that fat may constitute an additional basic taste group triggered by the trigeminal instead of the gustatory system [44–45]. However, while fat content may have affected the pleasantness of the gustatory stimuli, previous investigations specifically found that fat content did not affect auditory associations of pitch and timbre from gustatory stimuli [11]. Furthermore, by using complex flavored stimuli, we may not have elicited the most intense or apparent crossmodal associations as if we had used basic tastes. Finally, due to the complexity of the auditory stimuli, we have yet to identify which specific acoustical factors might be most predictive of crossmodal associations. Nevertheless, by using complex sound and taste we demonstrate the success of crossmodal associations, and the role of subjective preference as a possible mediating factor, in more ecologically valid perceptual experiences.
